# Urgent care centers in the U.S.: Findings from a national survey

**DOI:** 10.1186/1472-6963-9-79

**Published:** 2009-05-15

**Authors:** Robin M Weinick, Steffanie J Bristol, Catherine M DesRoches

**Affiliations:** 1 RAND, Arlington, Virginia, USA; 2 Institute for Health Policy, Massachusetts General Hospital, Boston, Massachusetts, USA; 3 Harvard Medical School, Boston, Massachusetts, USA

## Abstract

**Background:**

Due to long waits for primary care appointments and extended emergency department wait times, newer sites for episodic primary care services, such as urgent care centers, have developed. However, little is known about these centers. The purpose of this study is to provide information about the organization and functioning of urgent care centers based on a nationally representative U.S. sample.

**Methods:**

We conducted a mail survey with telephone follow-up of urgent care centers identified via health insurers' websites, internet searches, and a trade association mailing list. Descriptive statistics are presented.

**Results:**

Urgent care centers are open beyond typical office hours, and their scope of services is broader than that of many primary care offices. While these characteristics are similar to hospital emergency departments, such centers employ significant numbers of family physicians. The payer distribution is similar to that of primary care, and physicians' average salaries are comparable to those for family physicians overall. Urgent care centers report early adoption of electronic health records, though our findings are qualified by a lack of strictly comparable data.

**Conclusion:**

While their hours and scope of services reflect some characteristics of emergency departments, urgent care centers are in many ways similar to family medicine practices. As the health care system evolves to cope with expanding demands in the face of limited resources, it is unclear how patients with episodic care needs will be treated, and what role urgent care centers will play in their care.

## Background

With long waits for appointments with primary care providers, difficulty with same-day access for sick care, limited access to after-hours care, and extended emergency department waiting times, this decade has seen the growth of newer sites for the provision of episodic primary care services in the U.S. [1-8]. Driven by patients' willingness to seek care at alternative locations, retail clinics and urgent care centers have seen significant growth over the last decade [9,10]. Given their extended hours, availability of unscheduled appointments, and the range of services they provide, urgent care centers are uniquely positioned within the health care system to address the overflow of acute care patients from primary care as well as low- to mid-acuity emergency department patients [9].

Recent research has described the utilization of services and clinical content of care for retail clinics [11,12]. Other work has demonstrated that urgent care centers can decrease non-urgent emergency department use without a concomitant increase in hospitalizations; that urgent care center patient populations tend to look more like those in physician offices than in emergency departments; that these centers are busiest during the winter months; and that they can be more cost-effective for providing urgent care than an emergency department [13-16]. In addition, we previously found that approximately two-thirds of urgent care centers have been in operation for five or more years, and slightly more than half are physician-owned [17].

Despite this, the research base on urgent care centers in the U.S. has been lacking, with prior studies having typically been conducted in single urgent care centers. To date, there has been little information available about urgent care centers based on a nationally representative sample. In this paper, we describe the results of a recent survey of urgent care centers that is designed to understand how they are organized and how they function in the health care system. Throughout this paper we define urgent care centers in a manner developed in conjunction with the Urgent Care Association of America and consistent with prior definitions [9,17]. This includes those health care organizations that are not emergency departments, but typically (a) provide care primarily on a walk-in basis; are open (b) every evening Monday through Friday and (c) at least one day over the weekend; (d) provide suturing for minor lacerations, and (e) provide onsite x-rays.

## Methods

The sampling frame for this study was developed between September and November 2007 from three sources. First, we searched the website of each state's health insurance commissioner as well as America's Health Insurance Plans to identify all health insurance carriers doing business in every state. Each insurance carrier's website was then searched to identify any urgent care centers having contracts or referral arrangements with that carrier. Second, we searched three internet phone directories (, , and ) using a variety of terms such as "urgent care," "urgent care center," "walk-in clinic," and "drop-in clinic," and retained only relevant listings. Third, we obtained the mailing list for the Urgent Care Association of America, a membership-based trade organization dedicated to urgent care. These lists were de-duplicated to form the sampling frame. Through this process, we identified approximately 8,100 urgent care centers, with a likely undercount of centers that are part of hospitals (additional details are available [17]).

To ensure geographic diversity in the sample, urgent care centers were then selected at random within the four U.S. Census regions. The survey was conducted by mail with telephone follow-up between January and March 2008. The survey included questions on a wide range of topics, such as services provided, hours of operation, connections to other sectors of the health care system, use of health information technology, staffing, and financial data.

Prior to completing the survey, all organizations were screened to ensure that they were urgent care centers. This consisted of a series of five questions on the survey to assess whether the organization met each of the criteria included in the definition of an urgent care center (items (a) through (e) above). Any organization that did not meet all five criteria was not considered to be an urgent care center. Of the 1,703 sampled organizations, we received responses from 436 eligible urgent care centers. Two hundred and fifty seven urgent care centers refused to answer the survey. An additional 595 (34.9% of the total sample) responded that they were not urgent care centers, establishing them as ineligible for the survey. Following Response Rate 3 from the American Association for Public Opinion Research *Standard Definitions*, we applied an eligibility rate to those organizations we were unable to reach (n = 415) [18]. The eligibility rate represents the estimated proportion of cases of unknown eligibility that are actually eligible for the survey (e = total number of completed surveys/(total number of completed surveys + total number of ineligibles). Applying the eligibility estimate to our data resulted in a response rate of 50.2%. The surveys were most commonly answered by physicians (48.8%) and office managers (32.5%), with the remainder being completed by other clinical (11.9%) or non-clinical (6.9%) staff. This project was approved by the Partners Human Research Committee.

## Results

Table 1 shows urgent care centers' hours of operation, which are significantly expanded beyond typical nine-to-five office hours. More than two-thirds of urgent care centers open prior to 9:00 am during the week, with significant proportions doing so on Saturday (45.7%) and Sunday (31.1%). In addition, the majority of centers remain open until 7:00 pm or later on weeknights (90.6%), with two out of five remaining open until 9:00 pm or later. Approximately four in ten centers also remain open until 7:00 pm or later on Saturdays (40.9%) and one in three do so on Sundays (34.1%).

**Table 1:** Hours of operation

	**Typical opening time** Percent* (standard error)		
	
	**Before 8:00 am**	**8:00–8:59 am**	**9:00 am or later**
Weekdays (n = 428)	18.9 (1.9)	48.4 (2.4)	32.7 (2.3)
Saturdays (n = 425)	11.3 (1.5)	34.4 (2.3)	54.4 (2.4)
Sundays** (n = 418)	8.4 (1.4)	22.7 (2.1)	54.8 (2.4)

	**Typical closing time** Percent* (standard error)		
	
	**Before 7:00 pm**	**7:00–8:59 pm**	**9:00 pm or later**

Weekdays (n = 428)	9.3 (1.4)	49.5 (2.4)	41.1 (2.4)
	
	**Before 5:00 pm**	**5:00–6:59 pm**	**7:00 pm or later**
	
Saturdays (n = 423)	29.3 (2.2)	29.8 (2.2)	40.9 (2.4)
Sundays** (n = 419)	20.0 (2.0)	31.7 (2.3)	34.1 (2.3)

* May not add to 100% due to rounding.

** Does not add to 100% because 14.9% of centers report being closed on Sundays.

Staffing patterns are shown in Table 2. On average, each urgent care center with physicians (more than 95 percent of all centers) has 4.8 on staff, with 1.8 working full time. Notably, family physicians are the specialty that most commonly provide care at urgent care centers. They are present at nearly three-quarters of all centers, with an average of 3.3 family physicians on staff at those locations where they work (2.2 of whom work there full time). Fewer physicians trained in emergency medicine, internal medicine, pediatrics, and other specialties provide care at urgent care centers. Approximately half of all centers employ nurse practitioners and physician assistants (2.4 on staff on average if there is at least one), and slightly fewer than half employ at least one registered nurse (47.9%). Four out of five urgent care centers employ at least one medical assistant or other clinical staff member, with an average of 5.3 such employees at those centers that do use them (2.8 full time).

**Table 2:** Staffing

	**Centers with at least one on staff**	**Number on staff if at least one**		**Number on staff full time if at least one**	
	
**Type of clinician**	**%**	**Mean**	**Standard error**	**Mean**	**Standard error**
Physicians					
All specialties	95.8	4.8	0.2	1.8	0.1
Family practice	74.5	3.3	0.1	2.2	0.1
Emergency medicine	46.7	2.2	0.1	1.5	0.1
Internal medicine	33.8	1.5	0.1	1.3	0.1
Pediatrics	9.6	1.3	0.1	*	*
Other specialties	19.3	1.3	0.1	*	*
Nurse practitioners and physician assistants	52.9	2.4	0.1	0.8	0.1
Registered nurses	47.9	3.5	0.2	1.3	0.1
Medical assistants and other clinical staff	80.5	5.3	0.2	2.8	0.2

* No centers in our survey reported having physicians in these specialties who were on staff full time.

On average, urgent care centers saw 314 patients during the week preceding their response to the survey (Table 3), resulting in an average of 65.4 patients per urgent care physician per week. This is slightly lower than the national average of 84.4 visits per family physician per week, though comparable to the figure for family physicians in some regions of the country (e.g., 63.9 per week in the Mountain region) [19]. Approximately one in five urgent care centers have more than 450 patient visits per week (21.7%).

**Table 3:** Patient volume

**Number of visits per week**	**Percent of Centers**
0 – 149	15.8
150 – 299	33.7
300 – 449	28.8
450+	21.7
Mean number of visits per week (standard error)	314 (8.7)

In addition to suturing lacerations and providing onsite x-rays (required to meet our definition for inclusion in the survey), urgent care centers provide a wide variety of services (see Table 4). Occupational medicine is a significant component of the services provided by many centers, with more than nine in ten centers providing such services. Workers compensation evaluation and case management (37.2%) are substantially less likely to be provided than other occupational medicine services such as employment-related physicals, drug testing, and treatment of illness and injury.

**Table 4:** Services provided by urgent care centers*

	**Percent providing services**	**Standard error**
*Occupational medicine*		
Any occupational medicine services	92.6	1.3
Employment physicals	74.0	2.1
Employment-related drug testing	69.3	2.2
Treatment of workplace illness or injury	89.8	1.5
Case management and evaluation	37.2	2.3
*Lab tests processed onsite*		
Any lab tests processed onsite	93.3	1.2
Lab tests waived under CLIA**	87.2	1.6
Lab tests designated as moderate under CLIA	37.0	2.3
Tests requiring full laboratory certification	21.4	2.0
*Other diagnostic tests performed onsite*		
CT scans	14.0	1.7
Ultrasound	18.6	1.9
*Orthopedic-related services*		
Fracture care, including splinting and casting	80.7	1.9
Physical therapy and rehabilitation	20.7	2.0
*Medications*		
Prepackaged pharmaceuticals	48.6	2.4
Pain management	37.2	2.3
*Other treatments and services*		
Intravenous fluids	70.9	2.2
Primary care	54.4	2.4
Routine immunizations	63.5	2.3
Travel medicine services	32.6	2.3
Sports and school physicals	79.3	2.0

* n = 430 for all rows

** Clinical Laboratory Improvement Amendments

The large majority of centers provide onsite laboratory tests that are waived under the Clinical Laboratory Improvement Amendments (CLIA) (87.2%), with nearly two in five providing tests designated as moderate under CLIA, and 1 in 5 performing tests onsite that require full laboratory certification. Tests that are waived under CLIA are simple laboratory tests with a low risk of error as determined by the U.S. Food and Drug Administration, such as urinalysis by dipstick to check glucose levels. Tests designated as moderate are those with greater complexity and level of error than waived tests, but are not so complex as to require full laboratory certification [20,21].

While other diagnostic testing such as CT scans and ultrasounds are comparatively rare at urgent care centers, many centers provide a wide variety of other services. These commonly include fracture care (provided by 4 out of 5 urgent care centers), pain management (including prescribing and/or dispensing medications to manage acute and/or chronic pain), primary care, immunizations, and routine school and sports physicals. Seven in ten urgent care centers can provide intravenous fluids when needed. In addition, nearly half of urgent care centers (48.6%) provide prescription pharmaceuticals that are pre-packaged for dispensing a full course of treatment in doctors' offices rather than in pharmacies ("point-of-care" dispensing).

Table 5 shows relevant financial data for urgent care centers and comparable data for primary care and emergency departments where available from the published literature. At approximately $103, the average reimbursement per patient visit closely matches that for general/family practice and internal medicine office-based visits ($101) and is well below the mean payment for emergency department visits ($560) [22,23]. Even when compared against emergency department visits with no special services such as surgery or advanced imaging (mean $302), urgent care reimbursements are significantly lower; in fact, such reimbursements are below the 25^th ^percentile for emergency department reimbursements ($126) [23]. The distribution of primary payers looks markedly more similar to the overall payer distribution for primary care visits nationally than to the distribution seen in emergency departments, with approximately half of all urgent care visits (50.8%) having a private insurer as the primary payer [5,24].

**Table 5:** Reimbursement, payer distribution, and physician salary

	**Urgent care centers**		Comparison data*	
	
			**Primary care**	**Emergency departments**
			
	**Mean**	**Standard error**	**Mean**	**Mean**
Average reimbursement per patient (n = 209)	$102.96	$3.05	$101	$560
	
	**Percent**	**Standard error**	**Percent**	Percent**

Average distribution of primary payers (n = 261)				
Private insurance	50.8	1.2	57.7^†^	39.7^†^
Medicare	14.5	0.6	18.2^†^	17.3^†^
Medicaid or other public coverage	9.9	0.7	10.0	25.5^†^
Self-pay or uninsured	12.1	0.5	5.4^†^	17.4^†^
Occupational medicine***	12.7	0.9	1.0^†^	1.8^†^
	
	**Mean**	**Standard error**	**Mean**	**Percent**

Average physician salary				
Per hour (n = 215)	$80.97	$1.52	not available	not available
Per year (n = 112)	$158,845	$4,281	$152,300****	not available

* Sources for comparison data are referenced in the body of the text.

** Total exceeds 100% because more than one source of payment may be reported per visit.

*** Includes employer contracts and workers compensation.

**** For family physicians only.

^† ^Significantly different from urgent care centers, p < 0.05 or better.

Finally, the average annual physician salary at urgent care centers is $158,845, or an average of $161,940 based on the hourly average salary, assuming 2000 hours per year (difference not significant). This benchmarks comparably to the average from the American Academy of Family Physicians Practice Profile Survey [25]. While no equivalent data are collected by an emergency medicine physicians' organization, other salary and compensation surveys and guidelines place emergency medicine physicians' salaries at substantially higher levels, making urgent care center physician salaries far more comparable to those of family physicians [26-28]. As is often the case with survey data, fewer respondents answered our questions regarding financial information as compared with other topics included on the survey [29].

Two out of every five urgent care centers use electronic prescription ordering systems, with substantially larger proportions using computerized systems for viewing lab and imaging results, collecting patient demographics, billing, condition and procedure coding, and clinical notes (see Table 6). A recently-defined minimum set of necessary functionalities to qualify as a basic electronic health record system includes having patient demographics, prescription ordering, laboratory and image viewing, clinical notes, patient problem lists, and electronic lists of medications taken by patients [30]. The last two items were not included in our survey, as they are intended to help clinicians with the long-term management of multiple or chronic conditions, and therefore have less relevance for urgent care centers. Omitting these two items from the definition, 22.3% of urgent care centers have a basic electronic health record (data not shown), compared with 11% of all practices with 4 to 5 physicians nationally – those of a size comparable to the average urgent care center [30]. By this measure, electronic health record adoption at urgent care centers appears more comparable nationally to significantly larger practices with 11 to 50 clinicians. However, our inability to include the remaining two items means that we likely overestimate the proportion of urgent care centers with an electronic health record meeting the definition of basic functionality.

**Table 6:** Use of health information technology

	**Percent**	**Standard error**
*Percent using a computerized system for*		
Prescription ordering (n = 424)	40.3	2.4
With warnings and contraindications provided (n = 168)	83.9	2.8
With prescriptions sent electronically to the pharmacy (n = 165)	59.4	3.8
Viewing lab results (n = 422)	70.6	2.2
With out-of-range levels highlighted (n = 268)	90.7	1.8
Viewing imaging results (n = 426)	59.9	2.4
Clinical pathways and guidelines (n = 391)	36.8	2.4
Patient demographics (n = 424)	80.0	1.9
Billing and claims management (n = 417)	88.7	1.6
Condition and procedure coding (n = 414)	72.5	2.2
Clinical notes (n = 423)	52.5	2.4

## Discussion

In this first national survey of urgent care centers, we find that such centers employ significant numbers of family physicians, and are slightly larger on average than office-based practices nationally. We also describe the extent to which these centers have hours of operation expanded significantly beyond typical office hours, and the extent to which their scope of services is broader than that provided in many primary care offices.

While these characteristics reflect some similarities to emergency departments, we find that in other areas – most notably reimbursements, primary payer distribution, and physicians' salaries – urgent care centers seem far more similar to office-based family medicine practices. In addition, we find that urgent care centers are somewhat ahead of the curve in terms of adopting electronic health records in their practices, although these findings are somewhat qualified by our lack of precisely comparable data.

The interpretation of our findings is also limited by our comparatively small sample size, with fewer than 500 urgent care centers responding to our survey. In addition, despite the careful construction of our sampling frame, we have no method for assessing the completeness with which we identified urgent care centers. The significant proportion of organizations sampled for our survey that were not urgent care centers is indicative of the difficulty of assembling a full list of all centers in the U.S., particularly in a fluid, high-growth industry such as urgent care. Finally, this study does not enable us to discuss the clinical content of care provided at urgent care centers, but rather only to describe how they are organized and managed.

## Conclusion

At this point, the impact of urgent care centers on health care utilization and costs remains unknown, as does their impact on continuity of care and other aspects of health care quality. A significant shortage of primary care physicians is predicted over the next two decades, and more than one quarter of family physicians are not currently accepting new Medicare fee-for-service patients [31-33]. At the same time, despite having fewer emergency departments nationally, demand for their services continues to grow [5]. Given this combination, demand for urgent care center services may increase as well. Urgent care centers may provide a cost-effective alternative to the use of the emergency department for some conditions, but their impact on relationships between patients and their primary care providers – and on the costs of primary care provision – remains to be seen [34]. As the U.S. health care system evolves to cope with the expanding demands of population growth and aging in the face of limited resources, it is unclear how patients in need of episodic care will be treated, and what role urgent care centers will ultimately play in their care.

## Abbreviations

(CLIA): Clinical Laboratory Improvement Amendments.

## Competing interests

The authors declare that they have no competing interests.

## Authors' contributions

RMW obtained funding for the study, led its design and analysis, and drafted the manuscript. SJB and CMD participated in the design and analysis of the study, and helped to draft the manuscript. All authors read and approved the final manuscript.

## Pre-publication history

The pre-publication history for this paper can be accessed here:


